# Methylmercury and the Developing Brain

**DOI:** 10.1289/ehp.10302

**Published:** 2007-08

**Authors:** Leonardo Trasande, Philip J. Landrigan, Clyde B. Schechter, Richard F. Bopp

**Affiliations:** Mount Sinai School of Medicine, New York, New York, E-mail: leo.trasande@mssm.edu; Albert Einstein College of Medicine, Bronx, New York; Rensselaer Polytechnic Institute, Troy, New York

We reported that prenatal exposure to methylmercury causes cognitive impairment in an estimated 316,588 children born in the United States each year, costing this nation $8.7 billion annually in lost productivity (Trasande et al. 2005). Each year, this exposure also causes an estimated 1,566 cases of mental retardation (Trasande et al. 2006). The principal (70%) source of the mercury that enters the bodies of American children is combustion of coal in electricity-generating plants.

In their reanalysis of our data, Griffiths et al. (2007) made a series of incorrect judgments and poorly considered assumptions, each of which diminishes the import of our findings. We note the following errors in their analysis:

First, Griffiths et al. (2007) incorrectly used a linear model to relate cognitive function to prenatal methylmercury exposure, despite the National Research Council’s (NRC) clear finding that a logarithmic model provides a better statistical fit. The NRC, in their examination of the Faroe Islands cohort study, the study on which they place greatest reliance, stated that “[b]ecause these calculations necessitate extrapolating to estimate the mean response at zero exposure level,” logarithmic models “lead to lower estimates of the Benchmark Dose (BMD) than linear or K-power models” (NRC 2000, p. 294).

Recent analyses of early childhood lead exposure further corroborate the validity of logarithmic models in representing subclinical dose–response relationships of neuro-developmental injury (Canfield et al. 2003).

Second, Griffiths et al. (2007) unwisely based their analysis on potentially biased data from the Seychelles cohort study. By contrast, our model (Trasande et al. 2005), like that of the NRC, is based primarily on Faroes data. We chose not to use Seychelles data because of concern that the tests of neurobehavioral function used there were not well validated for a non-American population and therefore may not have been sensitive to detect cognitive impairment (Landrigan and Goldman 2003).

Another major potential source of bias in the Seychelles study, not acknowledged by Griffiths et al. (2007), is that it fails to consider the potentially beneficial nutrients found in the fish-based diet of the Seychelles. These nutrients, omega-3 fatty acids in particular, may partially offset the toxicity of methylmercury. Indeed, if maternal fish intake is taken into account in the Seychelles cohort, as recently was done, the estimate of methylmercury toxicity increases (Budtz-Jorgensen et al. 2007).

Griffiths et al. (2007) cited previous meta-analyses of the Faroes, Seychelles, and New Zealand studies by Ryan (2005) in applying IQ decrements of 0.13–0.18 points/ppm hair mercury, but these are likely underestimates, and further invalidate the analysis of Griffiths et al.

Third, in attributing mercury deposition to sources of emission, Griffiths et al. (2007) relied inexplicably and without justification on a mathematical model that posits that only 16% of deposits are attributable to American sources. They ignored empiric data from the U.S. Environmental Protection Agency (EPA)-sponsored Steubenville study, which found that 80–90% of mercury emissions deposit within 30–50 miles of the source (U.S. EPA 2007); and from the Electric Power Research Institute, which estimated that 30% of mercury deposits are attributable to American sources (Seigneur et al. 2004).

Fourth, Griffiths et al. (2007) incorrectly assumed that reductions in mercury emissions from power plants do not result in any reduced levels of fish contamination until after 15 years. This is not correct. Reductions in power-plant emissions in 2008 will, in fact, begin immediately to minimize methylmercury body burden among children born to women in 2008, and the degree of reduction will increase further in subsequent years, perhaps through 2038, thus reducing the number of children damaged, the severity of the prenatal brain damage in these children, and the resulting economic burden.

Finally, Griffiths et al. (2007) incorrectly based their estimate of the economic value of a child’s social productivity on the 1992 Current Population Survey rather than on the currently available 2005 data set. This miscalculation substantially underestimates the economic impact of methylmercury on the developing brain. Viscusi and Aldy (2004) estimated that this value is currently on the order of $4–9 million/child, a value far greater than that used by Griffiths et al., and greater even than our estimate.
